# Tracheal Aspirate Levels of the Matricellular Protein SPARC Predict Development of Bronchopulmonary Dysplasia

**DOI:** 10.1371/journal.pone.0144122

**Published:** 2015-12-11

**Authors:** Antonia P. Popova, Tracy X. Cui, Niko Kaciroti, Adam M. Goldsmith, Marisa J. Linn, Gloria S. Pryhuber, Marc B. Hershenson

**Affiliations:** 1 Department of Pediatrics and Communicable Diseases, University of Michigan, Ann Arbor, Michigan, United States of America; 2 Department of Molecular and Integrative Physiology, University of Michigan, Ann Arbor, Michigan, United States of America; 3 Center for Computational Medicine and Bioinformatics, University of Michigan Medical School, Ann Arbor, Michigan, United States of America; 4 Department of Biostatistics, School of Public Health, University of Michigan, Ann Arbor, Michigan, United States of America; 5 Department of Pediatrics, University of Rochester, Rochester, New York, United States of America; University of Cincinnati, UNITED STATES

## Abstract

**Background:**

Isolation of tracheal aspirate mesenchymal stromal cells (MSCs) from premature infants has been associated with increased risk of bronchopulmonary dysplasia (BPD). MSCs show high levels of mRNAs encoding matricellular proteins, non-structural extracellular proteins that regulate cell-matrix interactions and participate in tissue remodeling. We hypothesized that lung matricellular protein expression predicts BPD development.

**Methods:**

We collected tracheal aspirates and MSCs from mechanically-ventilated premature infants during the first week of life. Tracheal aspirate and MSC-conditioned media were analyzed for seven matricellular proteins including SPARC (for Secreted Protein, Acidic, Rich in Cysteine, also called osteonectin) and normalized to secretory component of IgA. A multiple logistic regression model was used to determine whether tracheal aspirate matricellular protein levels were independent predictors of BPD or death, controlling for gestational age (GA) and birth weight (BW).

**Results:**

We collected aspirates from 89 babies (38 developed BPD, 16 died before 36 wks post-conceptual age). MSC-conditioned media showed no differences in matricellular protein abundance between cells from patients developing BPD and cells from patients who did not. However, SPARC levels were higher in tracheal aspirates from babies with an outcome of BPD or death (*p*<0.01). Further, our logistic model showed that tracheal aspirate SPARC (p<0.02) was an independent predictor of BPD/death. SPARC deposition was increased in the lungs of patients with BPD.

**Conclusions:**

In mechanically-ventilated premature infants, tracheal aspirate SPARC levels predicted development of BPD or death. Further study is needed to determine the value of SPARC as a biomarker or therapeutic target in BPD.

## Introduction

Improved survival of very premature infants has been accompanied by an increased incidence of bronchopulmonary dysplasia (BPD), a chronic pulmonary disease characterized by hypoalveolarization and a requirement for supplemental oxygen for months or years. Development of BPD is usually preceded by respiratory distress syndrome (RDS), a common pulmonary complication of premature birth due to lung immaturity, surfactant deficiency and lung inflammation. In the “new BPD”, there are larger and fewer alveoli, as well as poorly formed secondary crests, indicating interference with septation [1]. As early as four days after birth, alveolar septa are thickened with α-smooth muscle actin-, TGF-β-positive myofibroblasts which persist until the healing stage of BPD [2]. Together, these data suggest that BPD may result from abnormal alveolar myofibroblast migration and activation. Identifying factors promoting myofibroblastic differentiation and potentiating fibrosis early in the neonatal lungs of prematurely born infants can advance development of strategies for prevention, diagnosis and treatment of BPD.

We have isolated mesenchymal stromal cells from tracheal aspirates of premature infants undergoing mechanical ventilation for respiratory distress syndrome (RDS) in the first week of life [3]. Although we previously referred to these cells as stem cells based on their colony-forming potential, surface markers and differentiation potential [3], we did not thoroughly test the clonogenicity or self-renewal of these cells, and therefore now refer to them as mesenchymal stromal cells (MSCs). Isolation of MSCs from tracheal aspirates increases the relative risk of developing BPD by over 20-fold [4]. MSCs show gene expression pattern associated with myofibroblast progenitor cells with high levels of mRNAs encoding contractile and extracellular matrix proteins [5]. Microarray analysis of neonatal lung MSCs shows high expression levels of mRNAs encoding matricellular proteins including SPARC (for secreted protein, acidic, cysteine-rich, also called osteonectin), connective tissue growth factor (CTGF), periostin, thrombospondin 1 (THBS1), cysteine-rich angiogenic inducer 61 (CYR61), transforming growth factor-β-induced (TGFBI) and galectin 1 (Gal1).

Matricellular proteins are non-structural extracellular matrix proteins which function as modulators of cell-cell and cell-matrix interactions and are expressed during morphogenesis, development, tissue injury and tissue remodeling. Among other actions, these structurally unrelated, secreted proteins are required for a subset of TGF-β responses and participate in myofibroblastic differentiation (reviewed in [6–10]). SPARC, in particular, increases TGF-β expression in mesangial cells [11] and enhances TGF-β-mediated Smad2 signaling in cardiac fibroblasts [12]. In fetal lung fibroblasts, inhibition of SPARC attenuates TGF-β-induced integrin-linked kinase activation and hydrogen peroxide production [13]. Conversely, TGF-β stimulates the expression of different matricellular proteins, including SPARC [8, 13–20]. Thus, matricellular proteins and TGF-β engage each other in a reciprocal relationship, resulting in feed-forward loops that can lead to inappropriate TGF-β-mediated fibrosis. SPARC also binds to fibrillar collagens I, II, III, IV, and V in the extracellular matrix and basal lamina [21]. SPARC-null mice have smaller collagen fibrils [22] and decreased interstitial collagen [23–26], demonstrating the requirement of SPARC for collagen deposition.

In human and animal studies, SPARC, CTGF, periostin and THBS1 have been associated with fibrotic disease of the lung and other organs [13, 25, 27–30]. In neonatal mice, lung overexpression of TGF-β1 [31] and CTGF [32] each result in structural changes described in BPD. Our group has previously shown that periostin is required for hyperoxia induced hypoalveolarization and interstitial fibrosis in a mouse model of BPD [33]. CTGF and CYR61 expression is rapidly increased in response to ventilator-induced lung injury in preterm lambs, suggesting rapid initiation of lung remodeling and the potential role of matricellular proteins as early lung injury biomarkers [34]. Together, these data suggest that matricellular proteins play a role in BPD development.

Little is known about the role of matricellular proteins in the pathogenesis of human BPD. We hypothesized that MSC-conditioned media and tracheal aspirates from infants mechanically ventilated for RDS in the first week of life contain matricellular proteins, and that concentrations of matricellular proteins correlate with the development of BPD. Such correlations might help identify targets for biomarker development and therapeutic intervention.

## Methods

### Patients

We examined tracheal aspirates from infants admitted to the C.S. Mott Children’s Hospital Newborn Intensive Care Unit. Entry criteria included gestational age at birth ≤ 32 weeks, mechanical ventilation for respiratory distress, and age ≤ 7 days. Infants with neonatal sepsis during their first week of life were excluded. Chorioamnionitis and necrotizing enterocolitis were diagnosed on clinical grounds. The University of Michigan Medical School Institutional Review Board specifically approved this study under the protocol “Alveolar Mesenchymal Cells in Neonatal Lung Disease" (HUM00042069). Written informed consent from both parents was obtained.

### Tracheal aspirate collection and MSC culture

Aspirates were collected during routine tracheal suctioning of mechanically ventilated premature infants during the first week of life as described [3]. Specimens were centrifuged (1,200 × g for 5 min at 15°C) and supernatants stored at −80°C. The cell pellet was resuspended in 2 ml Dulbecco's modified essential medium supplemented with 10% fetal bovine serum, 1% penicillin–streptomycin, 1% l-glutamine, and 0.5% amphotericin B. Adherent cells were incubated at 37°C and in 5% CO2 and grown to confluence. Low passage cells (2 or 3) were studied. For experiments, cells were plated at 50–60% confluence in 35-mm and 100-mm dishes. Following 24 hour incubation in serum free media, MSC conditioned media was collected and stored at -80°C.

### Measurement of matricellular proteins levels

Protein levels of THBS1, CTGF, Gal1, CYR61 and TGFBI were measured by enzyme-linked immunosorbent assay (all from R&D Systems, Minneapolis, MN except CTGF from PeproTech, Rocky Hill, NJ). SPARC and periostin were measured by multiplex assay (EMD Millipore Corporation, Billerica, MA). To control for sample dilution, matricellular protein levels were normalized to the secretory component of immunoglobulin A (scIgA) (ALPCO, Salem, NH) [35].

### Immunohistochemistry of human lung tissue

Lung sections were obtained from the Strong Memorial Hospital Neonatal Lung Biorepository under a protocol approved by the University of Rochester Institutional Review Board. Written autopsy consent, which includes permission for the use of tissue and deidentified clinical data, was obtained from the parents. Specimens were obtained from three term infants who died of non-pulmonary causes within three days of life, one infant born at 26 weeks gestation who died at 13 days of age, and four infants with BPD who died between 36–45 weeks postmenstrual age. The diagnosis of BPD was based on prematurity, need for chronic respiratory support and supplemental oxygen, chest radiographs and tissue diagnosis. Sections were immunostained with rabbit anti-human SPARC (Abcam, Cambridge, MA), labeled with biotinylated anti-rabbit IgG (Vectastain Laboratories, Burlingame, CA) and developed using the Vectastain Elite ABC kit and diaminobenzidine (Sigma-Aldrich, St. Louis, MO).

### Data collection and statistical analysis

The primary outcome variable for this study was BPD or the competing outcome death prior to 36 weeks postmenstrual age [36–40]. BPD was defined as the need for supplemental oxygen to maintain an acceptable oxygen saturation by pulse oximeter at 36 weeks postmenstrual age [41]. Statistical analysis was performed using SAS software, version 9.4 (SAS Institute, Cary, NC). All data were described as mean±SD for normally distributed measures or median and interquartile range for non-normally distributed measures. Demographic and clinical data were compared by *t*-test or Wilcoxon-Mann-Whitney test for continuous variables, and Fisher’s exact test for categorical variables. *P-*values were considered statistically significant if <0.05. For comparative analysis, matricellular protein concentrations were normalized to scIgA. Also, we used a multiple logistic regression model to determine whether tracheal aspirate matricellular protein levels were independent predictors of BPD/death controlling for gestational age (GA) and birth weight (BW). Model performance was assessed using area under the receiver-operating characteristic (ROC) curve.

## Results

### Patient data

We collected 89 tracheal aspirates from 89 infants. Sixteen patients died before they could be classified as having BPD. From the 73 patients surviving beyond 36 weeks postmenstrual age, 38 patients developed BPD, including 4 patients who died after reaching 36 weeks postmenstrual age. We compared the demographics of infants with the combined outcomes of BPD or death to those who did not develop BPD [36–40]. BW and GA at birth were lower, and the number of surfactant doses, frequency of necrotizing enterocolitis and culture-proven late-onset sepsis were higher in the BPD or death group (Table 1, next page). There was no gender difference between the two groups. As we found previously [4], a higher percentage of the patients who developed BPD had MSCs isolated from their tracheal aspirates.

**Table 1:** Baseline characteristics of babies who developed BPD or died before 36 weeks postmenstrual age (BPD/death) and babies who did not develop BPD (No BPD).

	BPD/death (N = 54)	No BPD (N = 35)	P-value
Male gender, n (% of total)	31 (57)	22 (63)	0.66
Gestational Age, wks, median (IQR)	25.93 (2.93)	28.71 (2.58)	<0.0001
Birth weight, grams, median (IQR)	815 (266)	1170 (490)	<0.0001
Postnatal Age at Sample Collection, days, median (IQR)	2 (2)	1 (1)	<0.001
MSC+, n (% of total)	32 (84)	19 (54)	<0.01
Surfactant doses, mean ± SD	2.3 ± 0.8	1.7 ± 0.6	<0.0001
Suspected clinical chorioamnionitis, n (% total)	10 (19)	2 (6)	0.12
Necrotizing enterocolitis, n (% total)	21 (39)	5 (14)	<0.05
Culture-proven late-onset sepsis, n (% total)	18 (33)	3 (9)	<0.01
Vent days, median (IQR)	28 (37)	3 (9)	<0.0001
O2 days, median (IQR)	116 (225)	27 (35)	<0.0001

Definition of abbreviations: IQR, interquartile range; Vent days, days requiring mechanical ventilation; O2 days, days requiring supplemental oxygen.

Gestational age, birth weight, postnatal age at sample collection, vent days and O2 days were compared using Wilcoxon-Mann-Whitney test. Number of surfactant doses was compared using Student’s t-test. Gender, frequency of MSCs, suspected clinical chorioamnionitis, necrotizing enterocolitis and culture-proven late-onset sepsis were compared using Fisher’s exact test.

### Neonatal lung MSCs secrete matricellular proteins

We have previously reported that neonatal lung MSCs express high levels of mRNAs encoding THBS1, Gal1, SPARC, periostin and TGFBI [5]. Therefore, we sought to confirm that MSCs produce and secrete matricellular proteins. MSCs isolated from 24 patients were cultured overnight in serum free conditions. Supernatants were collected and examined for periostin, CTGF, THBS1, Gal1, SPARC, CYR61 and TGFBI. Table 2 displays the median and interquartile range concentrations of the matricellular proteins detected in cell culture supernatants. MSCs produced nanogram quantities of THBS1, SPARC, TGFBI, periostin and Gal1, and smaller levels of CYR61 and CTGF.

**Table 2:** Unstimulated neonatal lung MSCs secrete matricellular proteins (n = 24).

Matricellular protein	median (interquartile range)
THBS1, ng/ml	80.87 (139.7)
SPARC, ng/ml	71.84 (37.85)
TGFBI, ng/ml	52.25 (70.19)
Periostin, ng/ml	16.31 (18.59)
Gal1, ng/ml	5.072 (8.410)
CYR61, pg/ml	94.58 (106.3)
CTGF, pg/ml	55.37 (15.09)

### Matricellular protein concentrations in tracheal aspirates of premature infants with respiratory distress syndrome

Little is known about factors facilitating lung repair during acute neonatal RDS and their role in subsequent development of BPD. We hypothesized that tracheal aspirates from premature infants undergoing mechanical ventilation for RDS contain matricellular proteins. Nanogram quantities of SPARC, TGFBI, Gal1, THBS1 and periostin, and smaller levels of CYR61 and CTGF were detected in the tracheal aspirates, with the highest concentrations found for SPARC (median concentration, 127.10 ng/ml) and TGFBI (70.76 ng/ml) (Table 3).

**Table 3:** Matricellular protein concentrations of tracheal aspirates from premature infants requiring mechanical ventilation for RDS. The number of samples, *N*, analyzed for each matricellular protein is shown.

Matricellular protein	median (interquartile range)	*N*
SPARC, ng/ml	127.1 (287.0)	73
TGFBI, ng/ml	70.76 (193.2)	58
Gal1, ng/ml	11.65 (28.15)	66
THBS1, ng/ml	5.91 (10.84)	67
Periostin, ng/ml	3.63 (4.16)	73
CYR61, ng/ml	0.99 (1.58)	70
CTGF, ng/ml	0.20 (0.26)	22

**Fig 1:**
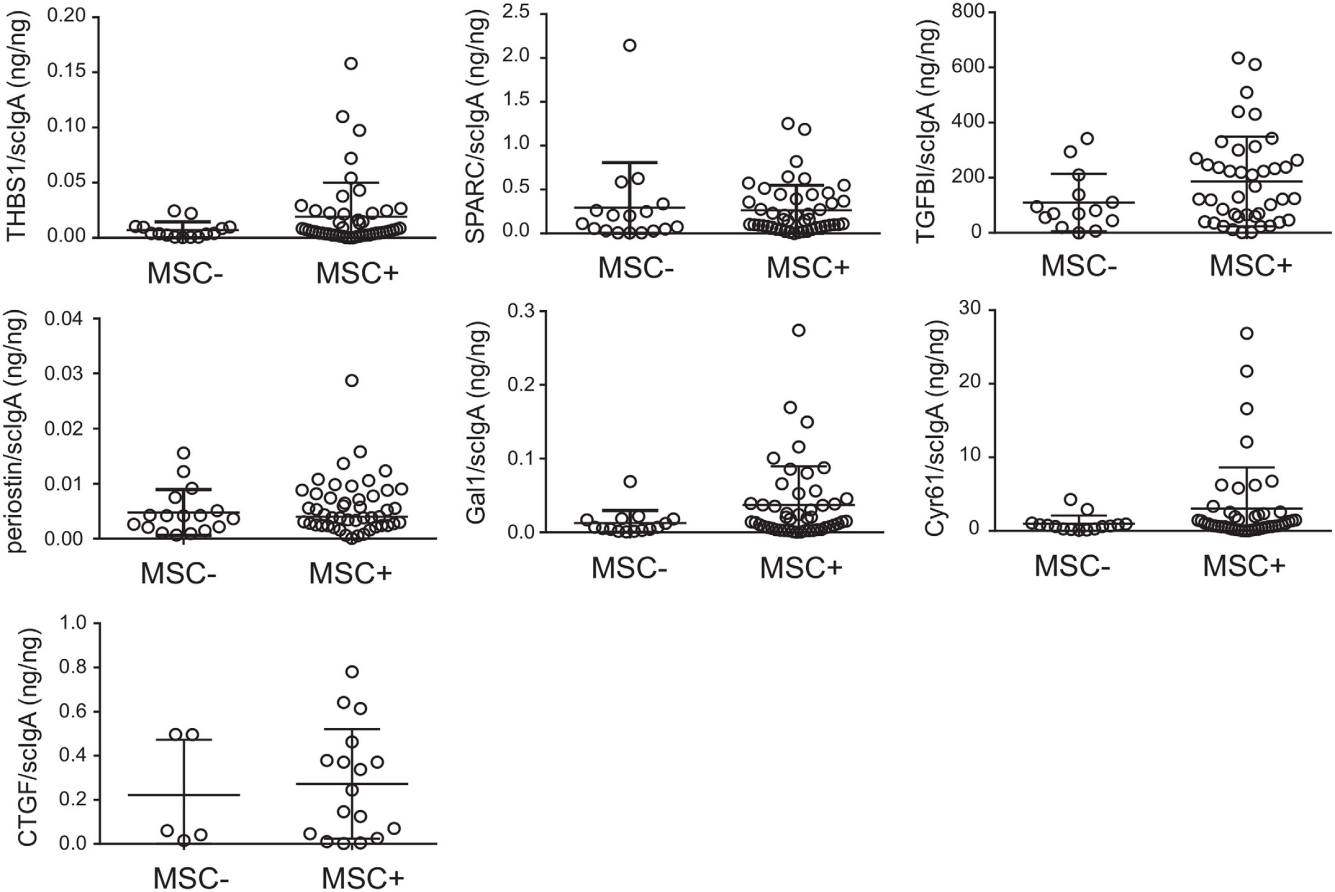
Mesenchymal stromal cell (MSC) isolation does not influence tracheal aspirate matricellular protein concentration. Tracheal aspirate matricellular protein and secretory component of IgA (scIgA) concentrations were measured by ELISA or multiplex immune assay. The isolation of MSCs from tracheal aspirates had no influence on matricellular protein concentration. The data show individual matricellular protein levels normalized to scIgA concentration. Medians and interquartile ranges are shown. Statistical significance was determined by Mann-Whitney U test.

To test whether MSCs might be a source of matricellular proteins in the tracheal aspirates, we compared the matricellular protein concentrations of tracheal aspirates from which MSCs were isolated to tracheal aspirates without MSCs (Fig 1). For comparative analysis, matricellular protein concentrations were normalized to scIgA. There were no significant differences in matricellular protein concentration between tracheal aspirates from which MSCs were isolated compared to aspirates in which cells were not isolated. Together these data suggest that there are other cellular sources of matricellular proteins in the lungs of premature infants undergoing mechanical ventilation for respiratory distress in the first week of life.

### SPARC levels predict development of BPD or death

**Fig 2:**
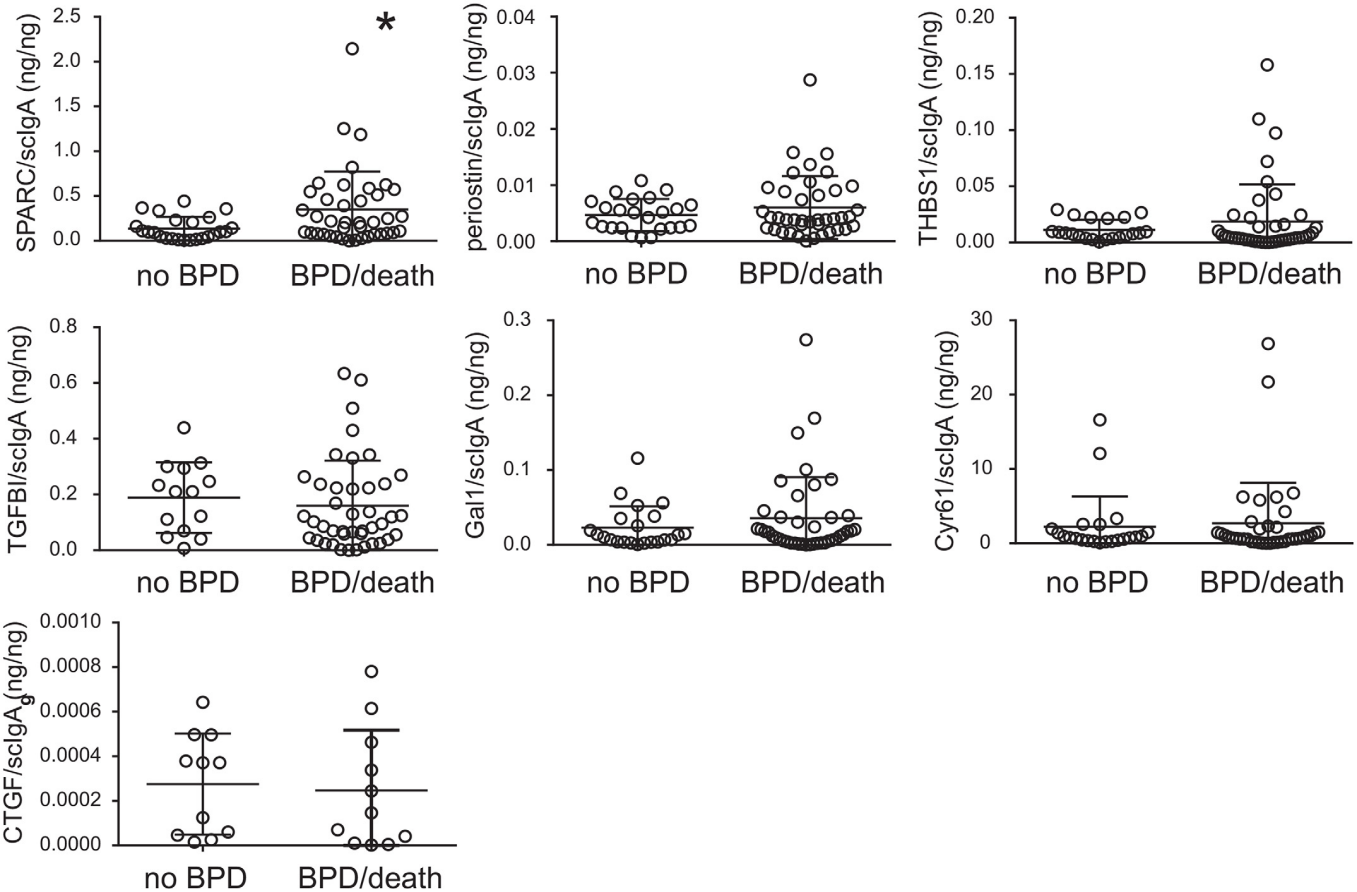
Normalized matricellular proteins and risk of developing BPD or death. Normalized SPARC levels were significantly higher in babies developing BPD or death prior to 36 weeks postmenstrual age (*p = 0.0185, Mann Whitney U test). There were no other significant differences in normalized matricellular protein concentration between infants who did not develop BPD and infants who developed BPD or died prior to 36 weeks postmenstrual age. Tracheal aspirate matricellular protein and secretory component of IgA (scIgA) concentrations were measured by ELISA or multiplex immune assay. The data show individual matricellular protein levels normalized to scIgA concentration. Medians and interquartile ranges are shown.

Matricellular proteins have been described as early markers of fibrosis in other diseases involving the lungs or other organs. We hypothesized that tracheal aspirate matricellular protein levels predict development of BPD or death before 36 weeks postmenstrual age. We compared matricellular protein levels in tracheal aspirates from infants who developed BPD or died before 36 weeks postmenstrual age and infants who did not develop BPD. Tracheal aspirate levels of SPARC were significantly higher in infants who went on to develop BPD or died before 36 weeks postmenstrual age than those who did not develop BPD (Table 4, next page). Normalized SPARC levels (SPARC/scIgA) were also significantly higher in the tracheal aspirates of infants who developed BPD or died before 36 weeks postmenstrual age (Fig 2). Only one sample yielded a SPARC level above the assay detection limit (a baby who developed severe BPD). Medians and interquartile ranges were 0.09899 ng/ng (0.1978) for 0.2047 ng/ng (0.4649) for BPD/death (p = 0.0185, Mann Whitney U test). Finally, there were no significant differences in matricellular protein expression between MSCs from patients developing BPD and MSCs from patients who did not develop BPD (data not shown).

**Table 4:** Matricellular protein levels in tracheal aspirates of babies who developed BPD or died before 36 weeks postmenstural age (BPD/death) and the ones who did not develop BPD (No BPD). For each group, the number of samples, *N*, analyzed for each matricellular protein is shown.

	No BPD	*N*	BPD/death	*N*	P-value
SPARC (ng/ml)	85.30 (177.3)	28	206.0 (395.5)	45	0.03
Gal1 (ng/ml)	10.20 (17.13)	24	11.96 (30.72)	42	0.61
THBS1 (ng/ml)	7.300 (9.910)	14	5.290 (14.72)	44	0.45
Periostin (ng/ml)	3.760 (2.712)	28	3.570 (5.210)	45	0.82
TGFBI (ng/ml)	121.9 (203.3)	23	70.03 (187.9)	44	0.33
Cyr61 (pg/ml)	1290 (1633)	26	944.9 (1578)	44	0.60
CTGF (ng/ml)	0.2011 (0.2821)	11	0.2039 (0.4154)	11	0.65

Data are median (interquartile range). Comparisons were performed using the Mann-Whitney U test.

To determine the independent predictive value of normalized SPARC for the development of BPD or death, we performed multiple logistic regression analysis. This analysis took into account the factors of GA and BW. The regression model showed that GA (β = 1.11, p<0.001) and normalized SPARC (β = -5.43, p<0.02) were independent predictors of BPD, whereas BW was non-significant when GA was in the model. The regression model showed that GA (β = 1.11, p<0.001) and normalized SPARC (β = -5.43, p<0.02) were independent predictors of BPD/death, whereas BW was non-significant when GA was in the model.

**Fig 3:**
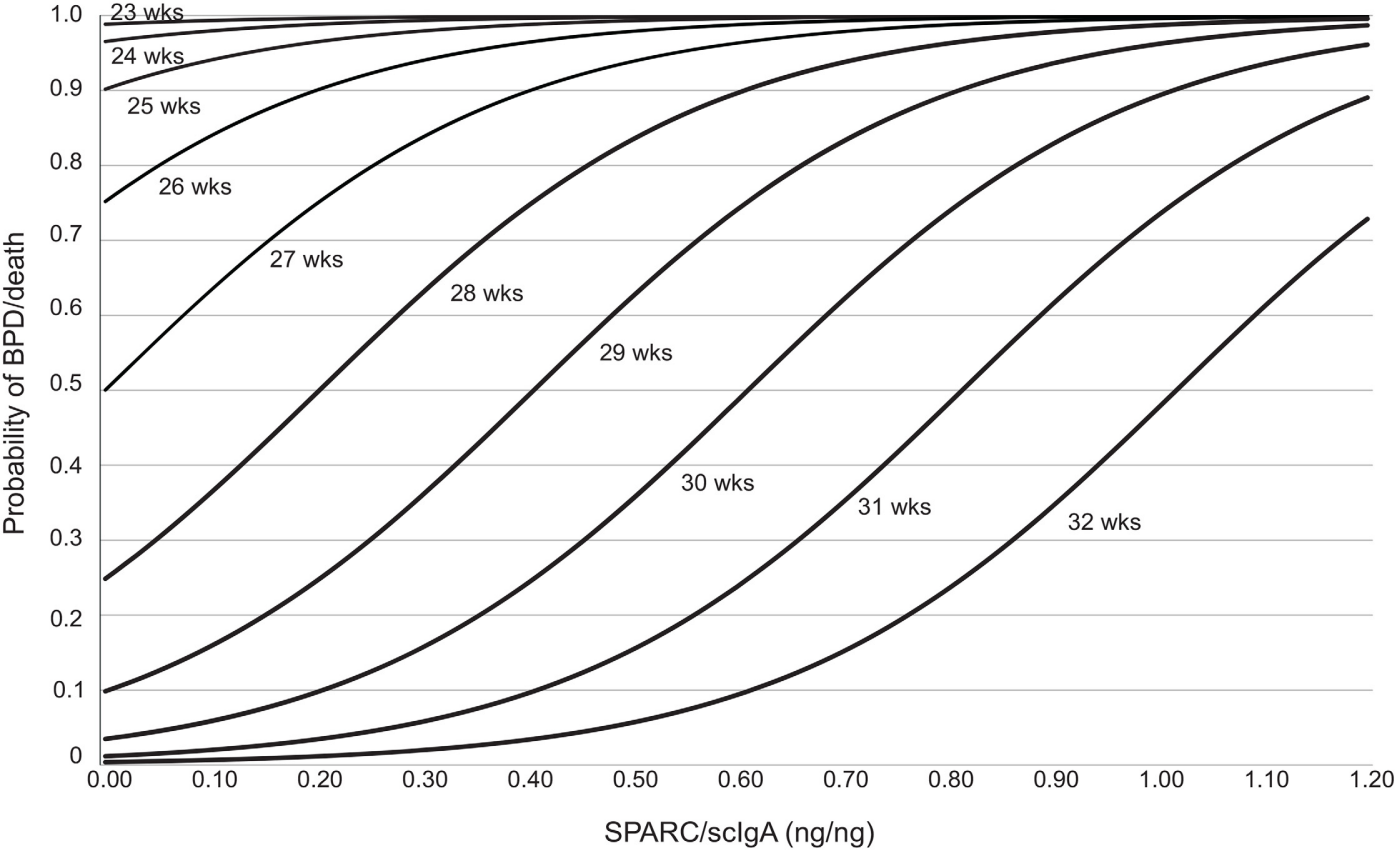
Probability of developing BPD or death as a function of normalized SPARC levels for different gestational ages. For gestational age (GA) between 26 and 32 weeks, low levels of normalized SPARC were protective for development of BPD or death. For the same gestational age, increasing levels of normalized SPARC were associated with an increased probability of BPD development of death before 36 weeks postmenstrual age. A GA less than 26 weeks had a high probability of developing BPD or death regardless of SPARC levels.

The area under the ROC curve was 0.86 for GA alone and 0.91 when normalized SPARC was added, indicating improved discriminate power for predicting BPD or death. We calculated the probability of developing BPD or death as a function of normalized SPARC levels for different GA (Fig 3). For GA between 26 and 32 weeks, low levels of normalized SPARC were protective for development of BPD or death, and increasing levels of normalized SPARC were associated with increased probability of development of BPD or death. GA less than 26 weeks had a high probability (>75%) of developing BPD or death, regardless of normalized SPARC levels. The odds ratios (OR) of developing BPD/death were OR = 0.33 (95%CI = 0.18–0.60) for one week increase in GA and OR = 1.75 (95%CI = 1.08–2.72) for 0.1 increase in normalized SPARC. In Table 5 (next page), the predictive power is summarized based on three categories of GA and two categories of normalized SPARC levels. Consistent with Fig 3, for infants born less than 26 weeks, the majority of the predictive power of developing BPD is driven by GA. For infants born between 26–29 weeks or older than 29 weeks, higher normalized SPARC levels improve the predictive power.

**Table 5:** Predictive power of developing BPD or death as a function of normalized SPARC for different GA.

	Gestational Age		
SPARC/scIgA	<26 weeks	26–29 weeks	>29 weeks
< = 0.3	100% (13/13)	47.4% (9/19)	0% (0/8)
> 0.3	100% (8/8)	100%(4/4)	40%(2/5)

### Increased SPARC expression in human lungs from infants with BPD

**Fig 4:**
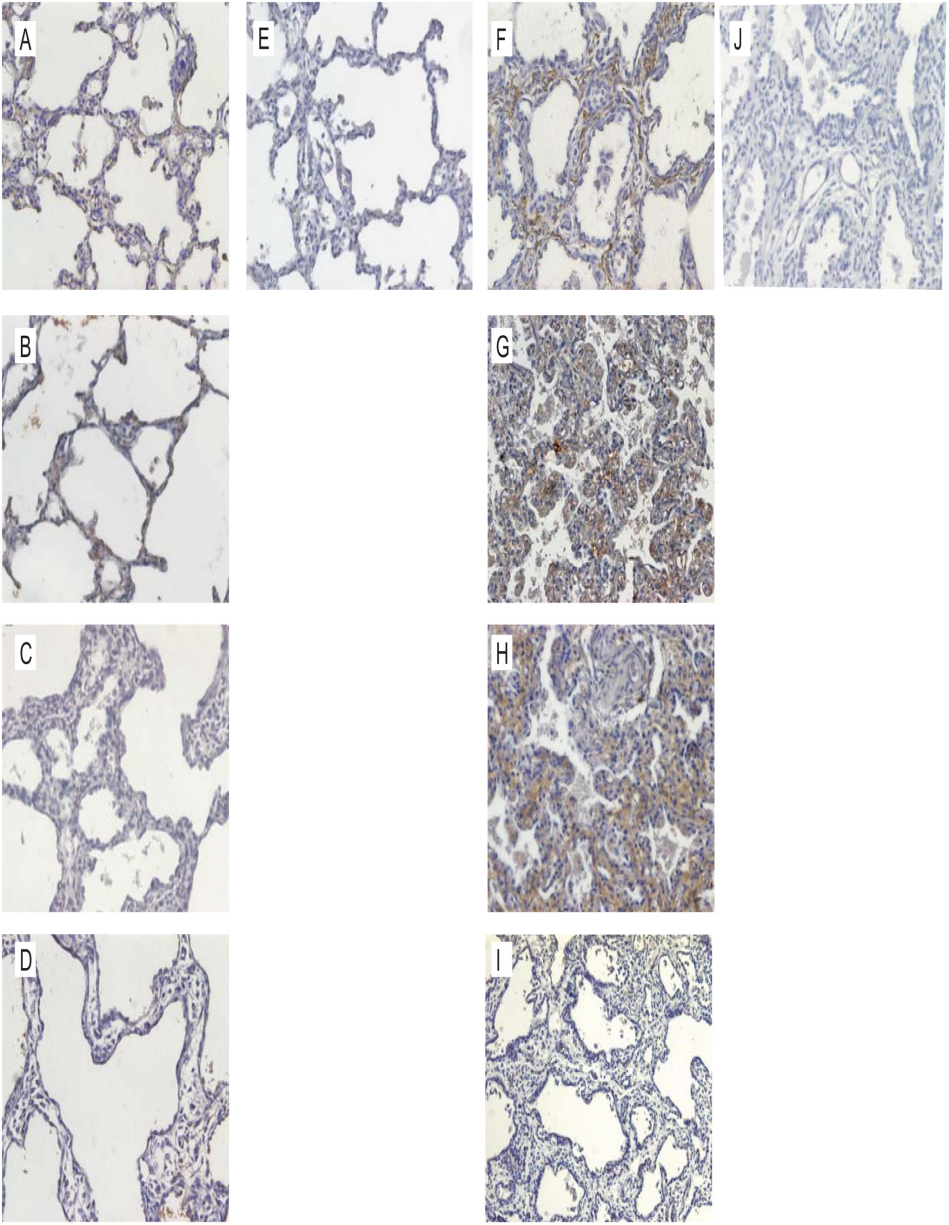
Increased SPARC expression in human lungs of infants with BPD. Lung sections were immunostained with rabbit anti-human SPARC, labeled with biotinylated anti-rabbit IgG and developed using the Vectastain Elite ABC kit and diaminobenzidine. A-C. Lungs of three full term infants dying of non-pulmonary causes are shown. There is little or no SPARC staining (brown) in the alveolar walls. D. The lung of an infant born at 26 weeks gestational age who died on postnatal day 13. Panel E is the IgG control for panel A. F-I. Lung sections from four infants dying of BPD. Three infants (F-H) show increased SPARC expression in the thickened alveolar interstitium. Panel J is the IgG control for panel F. (Original magnification, 200X).

Lung SPARC deposition of infants dying of BPD was compared with that from four infants without BPD, three full term infants and one born at 26 weeks gestational age (Fig 4). Minimal SPARC immunohistochemical staining of the non-BPD lungs was observed (Fig 4A–4E). The BPD lungs showed abnormal architecture, with thickened alveolar walls and wide alveolar spaces. Immunostaining for SPARC in BPD lungs was substantially increased in three of four infants, and was most prominent in the thickened alveolar interstitium (Fig 4F–4J).

## Discussion

Identification and quantification of the molecular components of tracheal aspirates from premature infants with RDS offers a window on processes occurring inside the distal lung soon after premature birth, including cell signaling and differentiation, gene expression patterns and protein synthesis, each of which can be measured *ex vivo* and help understand the mechanisms leading to the development of BPD. In this study we show that tracheal aspirates from premature mechanically ventilated infants in the first week of life contain nanogram quantities of matricellular proteins, including SPARC, TGFBI, Gal1, THBS1 and periostin, and smaller levels of CYR61 and CTGF. SPARC levels were higher in tracheal aspirates from infants who developed BPD or died before 36 weeks postmenstrual age. Normalized SPARC levels predicted development of BPD or death, independent of gestational age and birth weight. Furthermore, we showed that for gestational age between 26 and 32 weeks, increasing levels of normalized SPARC were associated with increased probability of development of BPD or death.

Various molecular and cellular factors have been implicated in the development of BPD, most notably the growth factors TGF-β and vascular endothelial growth factor (VEGF). Tracheal aspirate TGF-β1 levels are increased in preterm infants who develop BPD [42] and infants with RDS and BPD show the appearance of α-smooth muscle actin and TGF-β-positive myofibroblasts in the alveolar septa [2]. Lung overexpression of TGF-β results in a phenotype similar to BPD [31]. Lung VEGF expression is decreased in human infants dying with BPD [43] and recombinant human VEGF treatment enhances alveolarization after hyperoxic lung injury in neonatal animals [44]. Matricellular proteins may influence lung growth and the development of BPD via regulatory effects on TGF-β and VEGF. As noted above, matricellular proteins increase TGF-β-expression and activation and enhance TGF-β signaling [11–13, 45–47]. Conversely, TGF-β stimulates the expression of different matricellular proteins [8, 13–20], resulting in feed-forward loops that can lead to inappropriate TGF-β-mediated fibrosis. Matricellular proteins also modulate VEGF signaling, thereby regulating angiogenesis [48–52]. SPARC inhibits VEGF-induced proliferation of endothelial cells [51]. SPARC competitively inhibits binding of VEGF to its receptor and prevents phosphorylation of VEGFR1 and ERK1/2 [52].

In addition to its effects on TGF-β activation and signaling, SPARC also suppresses apoptosis of idiopathic pulmonary fibrosis fibroblasts through constitutive activation of β-catenin [53]. We have found that β-catenin signaling is activated in mesenchymal stromal cells from infants developing BPD [54]. We found similar changes in lungs of infants with BPD. Taken together, these results suggest that SPARC, perhaps through enhancement of TGF-β signaling and activation of β-catenin, contributes to fibrotic changes in the lungs of patients with BPD.

We previously reported that isolation of MSCs from tracheal aspirates of premature infants undergoing mechanical ventilation for respiratory distress syndrome in the first week of life is a potent biomarker for the development of BPD [4]. However, it should be noted that less than half the patients from whom MSCs were isolated developed BPD. We therefore hypothesized that other factors produced by the MSCs and present in the tracheal aspirates could be associated with the development of this disease. We found significant quantities of matricellular proteins in both tracheal aspirates and MSC-conditioned media. However, tracheal aspirate matricellular protein levels were not higher in tracheal aspirates from which MSCs were isolated, nor were there differences in matricellular protein expression between MSCs from patients developing BPD and MSCs from patients who did not. These data suggest that there are other sources of matricellular protein production besides MSCs. SPARC has a wide tissue distribution and is found not only in the connective tissue stroma of solid organs but is also expressed in macrophages at the sites of wound repair and cancer [55]. In our study, immunohistochemical stains showed SPARC localization in mesenchymal cells in the thickened alveolar interstitium of patients with BPD.

The primary outcome variable of our study was BPD or death prior to 36 weeks postmenstrual age. Patients dying prior to 36 weeks postmenstrual age are customarily combined with BPD [36–40] because, for prematurely-born infants, death before 36 weeks postmenstrual age is a competing outcome for BPD and may confound a cause-specific analysis. We repeated our analysis using BPD as the primary outcome variable and found similar results. Using a logistic regression model including normalized SPARC, GA and BW, both GA (p = 0.015) and normalized SPARC (p = 0.021) were independent predictors of BPD. In conclusion, we have shown that tracheal aspirates of mechanically ventilated premature infants in the first week of life contain matricellular proteins. Tracheal aspirate SPARC levels predicted development of BPD or death. When combined with GA, SPARC levels add additional sensitivity to predict BPD or death. We speculate that SPARC could be a therapeutic target for intervention against BPD. A prospective study is needed to determine the potential value of SPARC as a biomarker for the development of BPD.

## Supporting Information

S1 Dataset Demographic, clinical outcomes, raw matricellular protein concentrations and normalized matricellular protein concentrations data for infants from whom tracheal aspirates were collected. (XLSX)
